# Mind your heart: the epigenetic consequences of heart failure on brain function

**DOI:** 10.15252/emmm.202013785

**Published:** 2021-02-08

**Authors:** Gianluigi Condorelli, Michela Matteoli

**Affiliations:** ^1^ IRCCS Humanitas Research Hospital Rozzano Italy; ^2^ Department of Biomedical Sciences Humanitas University Pieve Emanuele Italy

**Keywords:** Cardiovascular System, Chromatin, Epigenetics, Genomics & Functional Genomics, Neuroscience

## Abstract

The bidirectional link between heart and brain has intrigued scientists for ages, but little is known on the underlying mechanism. In their recent study, Fischer and colleagues (Islam *et al*, 2021) propose a mechanism by which heart failure‐induced cognitive decline is linked to epigenetic changes that affect gene expression in neurons of hippocampus.

## Graphical abstract

The bidirectional link between heart and brain has intrigued scientists for ages, but little is known on the underlying mechanism. G. Condorelli and M. Matteoli highlight an epigenetic mechanism that may explain the heart‐failure induced cognitive decline (Islam *et al*, this issue of *EMBO Mol Med*).

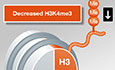

It is well known that cardiovascular disease may lead to behavioral changes. Indeed, the prevalence of depression and anxiety in patients with heart failure (HF) is 21% and 13–30%, respectively, the latter depending on the stringency of the diagnostic criteria; similarly, patients with depression and anxiety are associated with an approximately 20% increased risk of developing HF (Celano *et al*, 2018). Moreover, the incidence of Alzheimer’s disease and aging‐related dementia was suggested in the Framingham Study to be increased in patients with a low cardiac index (Jefferson *et al*, 2015). The problem is of great relevance, as HF affects approximately 25 million people worldwide and accounts for 1–2% of public healthcare costs in nations with “advanced” economies (Roger, 2013). Behavioral changes in the course of disease also mean less compliance with therapy and an increased need for assistance, further augmenting expenditures.

The mechanisms leading to behavioral changes in the course of HF are not known. The hypothesis that Islam *et al* develop is that cardiac dysfunction leads to modification of the transcriptional landscape in neurons of the hippocampus, a structure with a major role in memory and learning. The authors thus define whether and to what extent hippocampal neurons have a dysregulated gene expression profile in the course of HF. They used for most experiments a mouse model of cardiac‐specific overexpression of CamkIIδc, a kinase that normally controls Ca^2+^ metabolism and excitation–contraction coupling in cardiomyocytes by phosphorylating key substrates, but which if overexpressed leads to uncontrolled Ca^2+^ handing and gene expression, ending eventually in heart failure (Maier *et al*, 2003). Results were replicated, at least partially, using also a myocardial infarction mouse model of HF. Their results show that gene expression is profoundly affected in the hippocampal region upon the development of HF. In particular, the expression of oxidative and endoplasmic reticulum stress genes was altered in hippocampal neurons. Reduced perfusion of the brain, affecting also the hippocampus and related to a decreased cardiac function in HF, was suggested as the noxa sparking these changes in gene expression.

The authors then explored the mechanisms causing the modified gene expression in the hippocampus and uncovered that epigenetic regulation plays a significant role. Common histone modification marks are in dynamic equilibrium, and the levels of acetylation/deacetylation and methylation/demethylation of specific H3K residues depend on the activity of specific proteins: writers add acetyl or methyl groups, erasers play an opposite role, and readers translate the consequences of the covalent modifications into transcriptional changes (reviewed in ref. Greco & Condorelli, 2015). Incidentally, in HF development, epigenetics plays a critical role in gene expression modifications and the establishment of a disease phenotype (reviewed in ref. Papait *et al*, 2020). Islam et al here report that transcription associated with H3K4me3, a specific histone modification mark found on gene promoters exerting a global activating effect on transcription, is altered in the hippocampal cells of mice with CamkIIδc‐induced HF: Indeed, chromatin immunoprecipitation followed by DNA sequencing revealed that hippocampal genes marked with this histone modification are less expressed than in controls. Gene expression changes are associated with increased cellular stress pathways, leading to loss of neuronal euchromatin and reduced expression of a hippocampal gene cluster essential for cognition.

They also report that KMT2a, one of the two K4 methylases, is less expressed in hippocampal cells of mice undergoing HF. This finding links the modification of gene expression with reduced H3K4me3 deposition through decreased expression of this specific methylase. The relevance of KTM2a and H3K4me3 in mediating the consequences of HF on hippocampal gene expression and, consequently, on behavior is corroborated by previous report showing that H3K4me3 is critical for brain development and that altering this histone modification is associated with neurological diseases (Shen *et al*, 2014). Moreover, homologous recombination of the *Kmt2a* gene in mice was shown to increase anxiety and defective cognition (Jakovcevski *et al*, 2015).

The authors then determine whether increasing the level of H3K4me3 in hippocampal cells improves behavior in their model of HF. They followed a pharmacological approach, administering vorinostat—a drug used for refractory or relapsed cutaneous T‐cell lymphoma and currently tested in Alzheimer’s disease—which inhibits histone deacetylases and augments transcription, as histone acetylation is usually linked to the enhancement of transcription (Greco & Condorelli, 2015). The rationale for the use of this drug was that while it enhances histone acetylation, inhibiting repressive marks, it also increases methylation at K4 because of the interplay between histone acetylation marks and K4 methylation (Zhang *et al*, 2015). They show that administration of vorinostat improves the behavioral test scores of HF mice, concomitantly ameliorating gene expression and the level of H4Kme3 in hippocampal cells (Fig 1).

**Figure 1:**
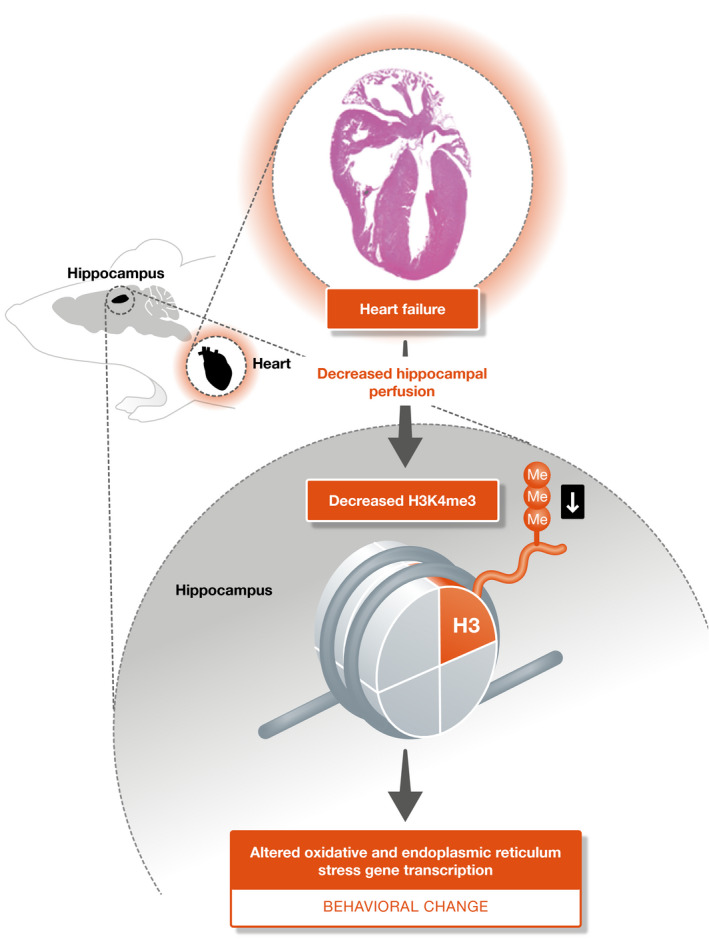
Heart failure is associated with decreased heart function, which leads hypoperfusion of the hippocampus This noxa triggers a decrease of H3K4me3 epigenetic modification mark, leading to reduced expression of a gene cluster essential for cognition.

The paper by Islam *et al* (2021) is of particular interest and novelty as it strongly suggests a link between HF and the central nervous system through epigenetic mechanisms regulating hippocampal function. It also suggests that vorinostat could be used for neurological disorders associated with HF. However, there are some challenging aspects. For example, the bulk of the experiments were conducted on an animal model of HF based on the overexpression of a kinase, a condition not encountered in physiology. Thus, data generated from other models of HF might further strengthen results of this study. Similarly, whether causes other than hypoperfusion could be responsible for changes of hippocampal gene expression during HF is a possibility which needs to be further explored. Another open question is whether histone marks other than H3K4me3 contribute to the modification of the hippocampal epigenetic landscape during HF. Lastly, it will be a stimulating challenge to determine whether histone modification drugs might modify hippocampal gene expression through systemic effects.

Results from this study clearly indicate that epigenetic mechanisms can underpin the behavioral modifications encountered in the course of cardiovascular disease, paving the way for future studies aiming at exploring therapeutic potential of these results and further strengthening the notion that brain and heart function are tightly linked.

## References

[emmm202013785-bib-0001] Celano CM , Villegas AC , Albanese AM , Gaggin HK , Huffman JC (2018) Depression and anxiety in heart failure: a review. Harv Rev Psychiatry 26: 175–184 29975336 10.1097/HRP.0000000000000162 PMC6042975

[emmm202013785-bib-0002] Greco CM , Condorelli G (2015) Epigenetic modifications and noncoding RNAs in cardiac hypertrophy and failure. Nat Rev Cardiol 12: 488–497 25962978 10.1038/nrcardio.2015.71

[emmm202013785-bib-0003] Islam MR , Lbik D , Sakib MS , Hofmann RM , Berulava T , Jiménez Mausbac M , Cha J , Goldberg M , Vakhtang E , Schiffmann C *et al* (2021) Epigenetic gene‐expression links heart failure to memory impairment. EMBO Mol Med 13:e11900 10.15252/emmm.201911900 PMC7933944 33471428

[emmm202013785-bib-0004] Jakovcevski M , Ruan H , Shen EY , Dincer A , Javidfar B , Ma Q , Peter CJ , Cheung I , Mitchell AC , Jiang Y *et al* (2015) Neuronal Kmt2a/Mll1 histone methyltransferase is essential for prefrontal synaptic plasticity and working memory. J Neurosci 35: 5097–5108 25834037 10.1523/JNEUROSCI.3004-14.2015 PMC4380991

[emmm202013785-bib-0005] Jefferson AL , Beiser AS , Himali JJ , Seshadri S , O'Donnell CJ , Manning WJ , Wolf PA , Au R , Benjamin EJ (2015) Low cardiac index is associated with incident dementia and Alzheimer disease: the Framingham Heart Study. Circulation 131: 1333–1339 25700178 10.1161/CIRCULATIONAHA.114.012438 PMC4398627

[emmm202013785-bib-0006] Maier LS , Zhang T , Chen L , DeSantiago J , Brown JH , Bers DM (2003) Transgenic CaMKIIdeltaC overexpression uniquely alters cardiac myocyte Ca^2^ ^+^ handling: reduced SR Ca^2^ ^+^ load and activated SR Ca^2^ ^+^ release. Circ Res 92: 904–911 12676813 10.1161/01.RES.0000069685.20258.F1

[emmm202013785-bib-0007] Papait R , Serio S , Condorelli G (2020) Role of the Epigenome in Heart Failure. Physiol Rev 100: 1753–1777 32326823 10.1152/physrev.00037.2019

[emmm202013785-bib-0008] Roger VL (2013) Epidemiology of heart failure. Circ Res 113: 646–659 23989710 10.1161/CIRCRESAHA.113.300268 PMC3806290

[emmm202013785-bib-0009] Shen E , Shulha H , Weng Z , Akbarian S (2014) Regulation of histone H3K4 methylation in brain development and disease. Philos Trans R Soc Lond B Biol Sci 369: 20130514 25135975 10.1098/rstb.2013.0514 PMC4142035

[emmm202013785-bib-0010] Zhang T , Cooper S , Brockdorff N (2015) The interplay of histone modifications – writers that read. EMBO Rep 16: 1467–1481 26474904 10.15252/embr.201540945 PMC4641500

